# Exercise as a Therapeutic Strategy for Obesity: Central and Peripheral Mechanisms

**DOI:** 10.3390/metabo14110589

**Published:** 2024-10-30

**Authors:** Yiyin Zhang, Ruwen Wang, Tiemin Liu, Ru Wang

**Affiliations:** 1 School of Exercise and Health, Shanghai University of Sport, Shanghai 200438, China; zhangyiyin1012@163.com (Y.Z.); wangruwen@sus.edu.cn (R.W.); 2 State Key Laboratory of Genetic Engineering, School of Life Sciences, Fudan University, Shanghai 200433, China

**Keywords:** obesity, exercise, central nervous system, thermogenesis, physical activity, adipose tissue, skeletal muscle

## Abstract

Obesity is a complex, multifactorial condition involving excessive fat accumulation due to an imbalance between energy intake and expenditure, with its global prevalence steadily rising. This condition significantly increases the risk of chronic diseases, including sarcopenia, type 2 diabetes, and cardiovascular diseases, highlighting the need for effective interventions. Exercise has emerged as a potent non-pharmacological approach to combat obesity, targeting both central and peripheral mechanisms that regulate metabolism, energy expenditure, and neurological functions. In the central nervous system, exercise influences appetite, mood, and cognitive functions by modulating the reward system and regulating appetite-controlling hormones to manage energy intake. Concurrently, exercise promotes thermogenesis in adipose tissue and regulates endocrine path-ways and key metabolic organs, such as skeletal muscle and the liver, to enhance fat oxidation and support energy balance. Despite advances in understanding exercise’s role in obesity, the precise interaction between the neurobiological and peripheral metabolic pathways remains underexplored, particularly in public health strategies. A better understanding of these interactions could inform more comprehensive obesity management approaches by addressing both central nervous system influences on behavior and peripheral metabolic regulation. This review synthesizes recent insights into these roles, highlighting potential therapeutic strategies targeting both systems for more effective obesity interventions.

## 1. Introduction

**Figure 1:**
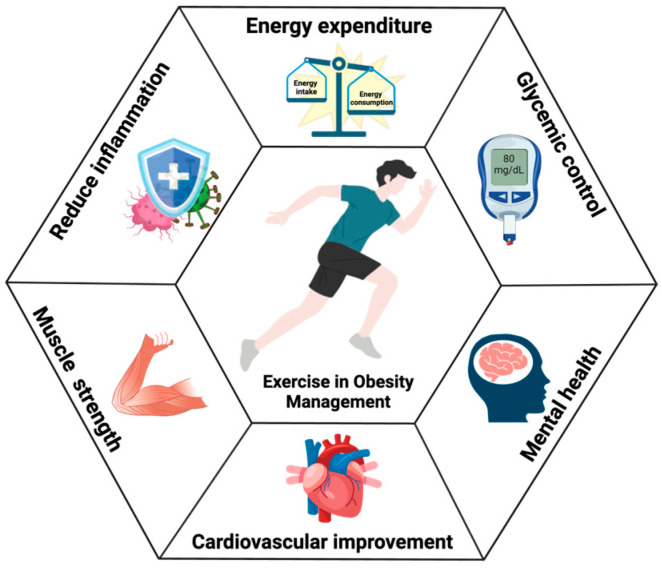
Overview of the physiological effects of exercise on various systems in relation to obesity management. The central figure represents physical activity, with surrounding icons illustrating exercise’s role in supporting (1) enhanced energy metabolism, (2) improved blood glucose regulation, (3) boosted cognitive function, (4) strengthened cardiovascular health, (5) increased muscle strength, and (6) enhanced immune response—all of which contribute to obesity prevention and reduction. “https://www.biorender.com/ (accessed on 23 October 2024)”.

Obesity, a complex illness with a growing incidence and associated problems, imposes costs on societies and economies all over the world [1]. According to a World Health Organization report in 2017, two billion adults (39%) were overweight and 650 million were obese (13%) around the world [2]. Obesity is a major risk factor of type 2 diabetes mellitus (T2DM) and skeletal muscle atrophy, and promotes the onset of metabolic and inflammatory dysfunction [3]. Obesity can be attenuated by decreasing energy intake and boosting energy expenditure. Currently, exercise intervention [4,5], a fasting intermittent diet (FMD), and gastrointestinal metabolic surgery are the most effective anti-obesity therapies [6,7]. However, a FMD should only be used as a therapy under the supervision of a physician, as adverse effects and problems can and do occur [8]. Using gastrointestinal metabolic surgery or medicines may cause side and potential harmful effects, even if the molecular mechanism is unclear [7,9]. Although exercise interventions have been proven to significantly reduce fat mass, promote energy expenditure, and improve metabolic health, many public health policies still lack sufficient support for exercise. Most global public health policies, such as the U.S. Centers for Disease Control and Prevention (CDC), primarily focus on dietary control and pharmacological interventions, with a limited emphasis on promoting exercise as a core intervention strategy [10]. This policy gap limits the potential of exercise interventions in the prevention and treatment of obesity. However, in obese humans, exercise aids in the reduction of fat mass, the acceleration of organic energy consumption, the inhibition of fat mass accumulation, and consequently the maintenance of body weight, while achieving a healthy metabolism at the same time (Figure 1). Exercise reduces fat mass and improves metabolic health through multiple mechanisms, including increased thermogenesis, hormonal regulation, and enhanced insulin sensitivity. Studies have demonstrated that regular exercise can significantly enhance thermogenesis by activating brown adipose tissue (BAT), thereby promoting fat oxidation [11,12]. In addition, exercise significantly regulates hormone levels related to fat metabolism, such as decreasing leptin levels and increasing adiponectin levels [13]. The reduction in leptin contributes to appetite suppression and decreased energy intake [14] and the increase in adiponectin levels promotes fatty acid oxidation and glucose metabolism, further enhancing fat breakdown [15]. Exercise also improves insulin sensitivity, enhancing glucose uptake by muscle cells and reducing the risk of insulin resistance, thereby helping to prevent excessive fat storage [16]. Consequently, exercise intervention is the top therapy for reversing obesity [4]. Strengthening policy support for exercise interventions will contribute to a more comprehensive approach in addressing the global obesity crisis.

Recent research indicates that exercise influences the formation and progression of obesity via the central nervous system’s metabolism and peripheral metabolism. Firstly, exercise can control appetite via the nervous system by changing food signals, inhibiting the cholinergic system, and regulating the reward system. In addition, exercise may improve cognitive function in overweight and obese persons by producing brain-derived neurotrophic factor (BDNF) [17], reducing neuroinflammation [18], and increasing cerebral oxygen absorption [19]. Secondly, exercise can regulate the homeostasis of the endocrine system through stimulating the secretion of leptin, insulin, ghrelin, and thyroid hormones. Thirdly, exercise promotes adipose tissue thermogenesis and secretes adipokines. Fourthly, exercise stimulates metabolic organs such as skeletal muscle and the liver to secrete myokines and hepatokines for anti-obesity. Fifthly, exercise improves the immune system in obesity. Although exercise controls the central nervous system, regulates the endocrine system, promotes adipose tissue thermogenesis, stimulates myokines and hepatokines, and enhances immune function, exercise biology has been virtually absent from public health. In this review, we will discuss these new insights and potential mechanisms and promising therapies to combat obesity, especially from the perspective of the central nervous system.

## 2. Introduction of Exercise Types Both in Human and Animals

### 2.1. Exercise Intervention in Human

Multiple studies show that exercise reduces body weight gain and fat mass, inhibits low-grade systemic inflammation, increases energy expenditure, and maintains a healthy metabolic fitness in obesity. The introduction and classification of exercise types used in obese humans are presented in Table 1. Aerobic exercise (AE) is a perfect method to improve aerobic capacity and health and plays a steady-state role in regulating energy production, blood flow, and matrix utilization [20,21,22]. Typical AE includes walking and running on a treadmill, yoga, tai chi, Pilates, and cycling. However, the AEs discussed in this review are walking and exercise on a treadmill. AE can be classified as low, moderate, high intensity, and very high intensity. Low-intensity AE typically corresponds to <55% of maximum heart rate (HRmax) or <40% of maximum oxygen uptake (VO_2_max). Moderate-intensity AE is defined as 55–74% HRmax or 40–69% VO_2_max. High-intensity AE is defined as 75–90% HRmax or 70–85% VO_2_max, while very high-intensity AE exceeds 90% HRmax or more than 85% VO_2_max [23].

Endurance exercise (EE), which is a form of high-intensity AE, has been shown to significantly improve cardiopulmonary function and metabolic adaptations, particularly under conditions of sustained high-intensity exercise [4,24,25]. High-intensity interval training (HIIT) is a type of exercise performed multiple times at anaerobic threshold or maximum lactate steady-state intensity for seconds to minutes. Research shows that although HIIT involves shorter total training times, it results in more significant metabolic improvements and fat reduction compared to traditional moderate-intensity continuous AE. This is mainly due to its higher post-exercise oxygen consumption and better metabolic adaptations [26]. In this review, HIIT is typically defined as having an intensity equal to or greater than 80% peak heart rate [27] or 90% VO_2_max or more [28].

The choice of exercise intensity should be adjusted based on an individual’s health status and exercise tolerance to achieve optimal health benefits. While HIIT has been shown to significantly improve metabolic function and reduce fat mass, its suitability can vary across populations. For younger individuals or older adults, HIIT offers clear benefits, particularly in terms of time efficiency and promoting metabolic adaptations [26,29,30]. However, in elderly individuals with cardiovascular diseases or other comorbidities, careful consideration must be given to the increased risk of falls and other exercise-related complications [31]. In these populations, moderate-intensity AE is generally a safer option, reducing cardiovascular stress while still providing substantial health benefits [29]. Additionally, low- to moderate-intensity AE can significantly improve cardiopulmonary function and metabolic health, making it particularly suitable for those who cannot tolerate high-intensity exercise [32].

### 2.2. Exercise Intervention in Rodents

Although exercise interventions in humans have demonstrated significant effects, their theoretical foundation and biological mechanisms are largely based on studies using animal models. Mice and rats, due to their high genetic homology with humans and their ability to closely simulate human physiology and pathology, provide valuable insights into the effects of exercise on metabolism, weight control, and immune function [33]. Mice are particularly favored in research due to their brief reproductive cycles, low cost, and well-established experimental protocols. These animal models offer reliable data for understanding the molecular mechanisms of exercise, aiding in the optimization of human exercise interventions.

The introduction and classification of exercise types used in obese rodents are presented in Table 2. All exercise intervention in rats and mice should be adapted at least 5–7 days before training. Treadmill training is the most common and effective intervention method in animal models, and its intensity can be artificially regulated by adjusting the speed or slope [27,33]. Treadmill training intensity can be determined by VO_2_max, maximal blood lactate, steady state, and maximal heart rate [33]. Different from treadmill exercises, voluntary wheel-running is an autonomous consciousness movement which reduces the stress levels of an animal. The distance of voluntary training can be monitored with tiny cameras or computers. Compared with the first two training types, resistant exercise (RE) is more complicated, in which mice climb ladders with heavy loads [34]. In order to achieve the desired effect, ladders are often set close to the vertical of the ground (gradient 80–85%) [35,36,37]. The intensity of the exercise was determined by the number of repetitions and the load on the tail. Swimming is also a common exercise intervention for animals. Water needs to be heated and kept at about 30 degrees before the formal intervention and animals need to be dried immediately after swimming. In addition, the protocol is divided into low intensity (20–60 min/day, 0~3% overload) [38], moderate intensity (61–89 min/day, 0~5% overload) [39], and high intensity (≥90 min/day, ≥0% overload) [40] according to the daily exercise time. Training time can also be adapted according to the aim.

## 3. The Role of Exercise in Obesity Intervention

### 3.1. Effect of Exercise on Appetite

Exercise reduces appetite through various mechanisms, a phenomenon widely validated by numerous studies. First, exercise can modulate the brain’s reward system, suppressing appetite and aiding in weight management. Chen et al. demonstrated that eight weeks of continuous treadmill training significantly increased dopamine (DA) levels in the nucleus accumbens and alleviated obesity [41]. However, individual sensitivity to DA regulation varies, which may lead to differences in appetite suppression effects [42]. Functional magnetic resonance imaging (fMRI) studies have shown that 60 min of high-intensity exercise can significantly inhibit activity in brain regions associated with hunger signals, thereby reducing appetite on a psychological level [43]. Cornier et al. further supports this view, finding that long-term exercise reduced the neural response to food cues, particularly in the insula, a region critical for the regulation of food intake, thereby contributing to weight loss and maintenance [44]. While these studies have revealed the impact of exercise on the perception of appetite cues, further research is needed to validate the role of different types of exercise in multisensory regulation.

In addition to modulating the brain’s reward system, exercise influences appetite by regulating hypothalamic metabolic mechanisms. Lactate metabolism has been shown to modulate pro-opiomelanocortin (POMC) and Neuropeptide Y (NPY) neurons in the hypothalamus and suppress appetite via the Jak2-STAT3 signaling pathway [45]. This mechanism is particularly evident in males, where high-intensity exercise activates POMC neurons, leading to appetite suppression. However, research has shown that sex differences reduce the appetite-suppressing effects of exercise. Studies indicate that in females, high-intensity exercise activates NPY/Agouti-related peptide (AgRP) neurons, which paradoxically increases appetite [46]. Previous research suggests that this may be due to women compensating for the energy deficit induced by exercise in order to preserve higher levels of body fat storage [47]. Therefore, exercise regimens should take sex differences into account and be personalized accordingly.

Exercise also suppresses appetite by regulating appetite-related hormones such as acylated ghrelin, peptide YY (PYY), and glucagon-like peptide-1 (GLP-1). Different types and intensities of exercise have varied effects on these hormones. Studies have shown that HIIT significantly reduces ghrelin levels while increasing concentrations of PYY and GLP-1, effectively suppressing appetite [48,49,50]. Notably, weight-bearing exercises demonstrate a more pronounced appetite-suppressing effect compared to non-weight-bearing exercises [51]. In contrast, brisk walking does not induce significant changes in appetite hormones, further validating the role of high-intensity exercise in weight management [52]. These studies indicate that exercise affects appetite not only through the nervous system but also by modulating the levels of appetite-related hormones, reinforcing the multiple benefits of exercise in weight management. However, when discussing the appetite-suppressing effects of different types of exercise, attention must be paid to the long-term effects of exercise intensity. Although high-intensity exercise can significantly suppress appetite in the short term, the increased energy expenditure may lead to heightened post-exercise hunger, potentially increasing food intake [53,54]. This highlights the balance between short-term and long-term energy needs. Therefore, when designing exercise-based weight loss programs, it is essential to consider both exercise intensity and individual metabolic demands to achieve more effective weight management and health maintenance. This further reinforces the multifaceted benefits of exercise in weight management while emphasizing the importance of individualized adjustments.

Lactate metabolism may be another key mechanism through which exercise regulates appetite. Studies have shown that lactate can inhibit the secretion of the hunger hormone ghrelin while increasing the levels of GLP-1 and PYY, thereby suppressing appetite [45,55]. However, different types of exercise have varying effects on lactate levels, and the impact of lactate metabolism on appetite regulation may differ significantly depending on the form of exercise, which requires further investigation.

Moreover, exercise influences appetite by modulating the levels of inflammatory factors such as IL-6. The increase in IL-6 levels following acute exercise is associated with a reduction in energy intake, suggesting that IL-6 may act as an anorexigenic factor [45,56]. Recent studies have also identified new metabolic byproducts, such as N-lactoyl-phenylalanine (Lac-Phe), which regulate energy balance by suppressing appetite and reducing obesity [57]. The roles of asprosin and BDNF in exercise have also garnered attention, particularly in obese individuals, where HIIT significantly reduces asprosin levels while increasing BDNF levels [57].

In conclusion, exercise reduces appetite through various mechanisms, including the regulation of the brain’s reward system, appetite hormones, lactate metabolism, and changes in inflammatory factors. These mechanisms work synergistically, underscoring the critical role of exercise in weight management and metabolic health. However, further research is needed to deepen our understanding of individual variability and long-term effects to optimize the practical application of exercise interventions.

### 3.2. Effect of Exercise on Thermoregulation

Thermoregulation is one of the key factors influencing exercise performance and health. Whether during short-term high-intensity exercise or long-term endurance training, the body relies on a series of complex physiological mechanisms to maintain core temperature balance. Thermoregulation depends not only on the heat production and dissipation mechanisms of skeletal muscles but also on the precise control by the central nervous system.

The rapid rise in body temperature primarily stems from the energy metabolism of skeletal muscles, which is a result of energy expenditure during high-intensity exercise. In the early stages of exercise, heat production in skeletal muscles relies mainly on phosphocreatine (PCr) hydrolysis and anaerobic metabolism. However, as exercise continues, aerobic metabolism gradually dominates the energy supply, leading to an increase in heat release [58,59]. As the exercise intensity increases, skeletal muscle heat production rises significantly and thermoregulatory mechanisms are correspondingly enhanced, with heat being transferred from the muscles to the body surface for dissipation through the circulatory and lymphatic systems [60]. At this point, the hypothalamus plays a crucial role in the central control of thermoregulation, coordinating the processes of heat production and dissipation throughout the body to ensure that the body temperature remains within an optimal range.

In addition to skeletal muscles, BAT also plays an important role in heat production during exercise. BAT increases heat generation to raise the core body temperature, aiding muscles in boosting strength and reducing muscle viscosity in the early stages of exercise, thereby accelerating the body’s adaptation to physical activity [11]. This process complements the heat production mechanism of skeletal muscles, working together to maintain thermal balance during exercise. However, as exercise continues, BAT thermogenesis gradually decreases [61]. Studies have shown that prolonged elevation of core temperature can lead to reduced BAT activity, and even a “whitening” phenomenon may occur [62]. Simultaneously, the hypothalamic regulatory mechanism shifts heat distribution to other adipose tissues, compensating for BAT function through a browning process, thereby maintaining overall energy balance.

The magnitude and duration of enhanced post-exercise oxygen consumption (EPOC) depend on the intensity and duration of exercise, with significant increases observed following high-intensity exercise. Research has shown that part of EPOC’s effect is due to energy consumption during the restoration of body temperature [63]. High-intensity exercise markedly raises the core temperature, prolonging the duration of EPOC [64,65]. During temperature recovery, metabolic activities such as ATP replenishment and lactate clearance contribute to increased energy expenditure [66]. Although EPOC accounts for a relatively small portion of overall energy expenditure, its mechanism of increasing energy consumption through temperature recovery, especially after high-intensity exercise, is of significant importance for weight control.

The regulation of body temperature relies not only on the heat production from skeletal muscles and BAT but also on the crucial role played by the central nervous system. The regulatory function of the hypothalamus is not limited to thermoregulation, but is closely linked to peripheral energy metabolism. For instance, the muscarinic cholinergic receptors and nitric oxide (NO) signaling pathways in the ventromedial hypothalamus (VMH) and paraventricular nucleus (PVN) also participate in thermoregulation. Inhibition of these pathways leads to increased heat storage and reduced heat dissipation, which accelerates fatigue [67,68]. Acute exercise enhances central nervous regulation of body temperature by activating the leptin-induced ERK1/2 pathway, which increases BAT thermogenesis and thereby boosts energy expenditure, particularly in obese individuals [68,69]. Additionally, environmental temperature during exercise can influence these regulatory mechanisms. The hypothalamus plays a key role in regulating body temperature during exercise, with DA and norepinephrine (NA) in the preoptic area (POA) of the hypothalamus modulating metabolic rate, delaying fatigue, and accelerating heat dissipation. This regulatory effect is particularly pronounced in high-temperature environments, where DA enhances exercise performance by improving heat tolerance and dissipation efficiency [70,71,72]. In contrast, in cold environments, the thermoregulatory mechanisms operate differently, adapting to conserve heat rather than dissipate it [73]. As exercise training continues, the body’s thermoregulatory capacity gradually improves, with a marked increase in adaptation to high-temperature environments. Upon receiving feedback from central temperature receptors and peripheral skin sensors, the hypothalamus triggers appropriate responses, such as increasing sweat production and skin blood flow, to dissipate excess body heat and maintain a stable core temperature, thereby improving exercise performance [74].

In summary, exercise increases body temperature through the activation of skeletal muscles and BAT, while thermoregulatory mechanisms dissipate excess heat via the circulatory and lymphatic systems. High-intensity exercise raises body temperature in the short term and enhances post-exercise oxygen consumption, thereby extending energy expenditure. The central nervous system plays a key role in maintaining thermal balance under varying conditions, improving heat tolerance during exercise, and supporting post-exercise energy expenditure, which contributes to weight management. While research has established a connection between exercise intensity and thermogenesis, the relationship between different types of exercise and thermoregulation remains underexplored. Understanding how various exercise modalities influence thermoregulation will provide valuable theoretical insights for optimizing exercise prescription design.

### 3.3. Effect of Exercise on Anxiety and Cognition

Cognitive dysfunction is one of the comorbidities in obesity and results from the synaptic remodeling, hypothalamic neurodegeneration, and the disruption of hypothalamic circuitry and hypothalamic outputs [75]. Neuronal mitochondria regulate the neuroplasticity through both adulthood and developmental stages [76]. Exercise increases the anti-oxidant capacity of mitochondria, the complexity of the electron-transport system, and the rate of biogenesis, as well as reducing the expression of oxidative damage and apoptosis markers [77,78], ultimately leading to a key adaptation of the mitochondria in hippocampal neurons that supports the increased metabolic demands [79].

### 3.4. Effect of Exercise on Adipose Tissue

Adipose tissue exerts an important role on whole-body energy expenditure, glucose and insulin hemostasis, hormonal regulation, and fat accumulation [80]. It mainly consists of white adipose tissue (WAT) and BAT. WAT is found throughout the whole body and divided into intro-abdominal or sub-cutaneous tissues. As the site for energy storage, WAT synthesizes triglycerides for storage and breaks down triglycerides into free fatty acids for supplying energy. WAT regulates energy metabolism by secreting adipokines such as leptin and adiponectin and supplying energy [81]. BAT is considered as a source of thermogenesis, which stimulates a healthy phenotype, benefits insulin sensitivity, and acts as a promising target for anti-obesity [82]. Studies shows that BAT could promote energy expenditure and fatty acid oxidation, ultimately reducing fat mass [83]. Excess accumulation of WAT will attribute to obesity, while BAT is a target for anti-obesity.

Exercise is a stimulator that promotes the browning of WAT, accelerates glycolipids, decreases the insulin requirement and suppress the occurrence and development of obesity [84]. Firstly, exercise upregulates the expression of thermogenic genes such as Prdm16 and Ucp1 in WAT, which increases the formation of lipid droplets and therefore increases non-shivering thermogenesis [85]. Secondly, exercise induces the secretion of myokines such as myostatin [86,87], irisin [88], fibroblast growth factor 21 (FGF21), or others [89], which stimulate the WAT beiging. Myostatin inhibits skeletal muscles and is negative with WAT beiging. Exercise improves insulin sensitivity and WAT beiging by inhibiting the secretion of myostatin. Irisin is a hormone-like myokine and acts on the WAT to activate the browning response once released into circulation. Exercise increases the irisin expression and therefore stimulates the browning [90]. FGF21 is a secreted, circulating hormone [91]; it can induce thermogenesis of BAT and WAT in mice. Exercise stimulates the secretion of FGF21 and therefore acts as an anti-obesity treatment. Thirdly, multiple key adipokines produced by fat govern the irisin-induced browning process in a paracrine way [92]. In leptin-deficient ob/ob mice, key markers of brown adipocyte morphology and function are diminished, demonstrating a crucial role for leptin in brown adipogenesis and non-shivering thermogenesis. In addition, leptin and insulin function synergistically on various POMC neuronal subpopulations to enhance WAT browning and energy expenditure, as well as to protect against diet-induced obesity [93]. In addition to the aforementioned two methods, a number of fat secretion variables can stimulate thermogenesis. Zinc-2-glycoprotein (zag) is a 43 kDa adipokine, and intraperitoneal injection of a zag expression plasmid decreases body weight and adiposity as well as induces fat browning in subcutaneous fat depots [94]. Adiponectin is one of the most common adipokines released by adipocytes, and the stimulation of M2 macrophage proliferation by adiponectin provides a novel way to cause cold-induced browning of subcutaneous adipose tissue [95]. Moreover, beige adipocytes release substances that can influence adipocyte and other organ activities. In this manner, beige adipocytes secrete Slit2, a member of the slit family of homologous proteins, to improve fat thermogenesis, increase energy expenditure, and induce the browning of inguinal tissue, which increases thermogenesis.

Different types of exercise have varying effects on the browning of WAT and activation of BAT. For instance, HIIT has been shown to significantly promote the browning of WAT, primarily due to the high metabolic demand and the triggering of inflammatory signaling [96,97,98]. In contrast, moderate-intensity continuous training promotes browning through inflammatory signaling, though its effects are less pronounced compared to those of HIIT [98]. Resistance training can also induce adipose tissue browning [99]. These differences highlight that exercise type and intensity should be carefully considered when aiming to optimize adipose tissue remodeling for weight management and metabolic health.

To summarize, adipose tissue plays a critical role in regulating energy metabolism, glucose and insulin homeostasis, hormonal balance, and fat accumulation throughout the body. Exercise promotes the browning of WAT, increasing energy expenditure and helping prevent obesity. It does so by upregulating the thermogenic gene expression in WAT and by inducing the secretion of key myokines, such as myostatin, irisin, and FGF21, which stimulate the browning process. Additionally, adipokines like leptin, insulin, and zinc-2-glycoprotein contribute to thermogenesis and fat browning, enhancing energy expenditure. These mechanisms work together to highlight the multifaceted benefits of exercise in obesity prevention and metabolic health. However, further research is required to fully understand how to optimize these mechanisms for effective obesity treatment.

### 3.5. Effect of Exercise on Liver

As a metabolic organ in the body, liver plays a major effect on glucose homeostasis through multiple pathways such as glycogenolysis, glycogenesis, gluconeogenesis, and glycolysis [100]. Dysfunction of the liver contributes to insulin resistance and fatty acid liver. In addition, liver is also considered as an organ that removes toxins, produces proteins, secretes bile and digests food, stores blood, and maintains hematopoiesis and defense immune function. Obesity is a multiple-factor disease and featured as excessive fat accumulation. Research shows that obesity leads to the disorder of liver function and induces liver-related diseases such as nonalcoholic fatty liver (NAFLD) or nonalcoholic steatohepatitis (NASH) [101]. Therefore, maintaining the health of the liver is helpful to the treatment of obesity and liver-related diseases such as NAFLD and NASH. Decades studies shows that exercise is an effective treatment to improve the liver function in obesity.

Exercise stimulates the secretion of hepatokines such as FGF21, follistatin, angiopoietin-like 4 (ANGPTL4), fetuin A, heat shock protein 72 (HSP72), and insulin-like growth factor binding protein 1 (IGFBP1) [102] and decreases fatty liver via the combined effects of those hepatokines. FGF21 is a stress-inducible hormone which exerts an important role in maintaining energy balance and glucose and lipid homeostasis [103]. Exercise stimulates the secretion of FGF21 both in circulation and the liver. For the liver, increased FGF21 promotes fatty acid oxidation and suppresses lipogenesis, which ultimately decreases the fat mass in the liver [104]. In addition, increased FGF21 in the liver could also make a cross-talk effect on adipose tissue and reduces the fat mass. Follistatin acts as a single-chain glycosylated protein and has a vital role in the regulation of liver homeostasis [105]. Circulating follistatin can continue to increase up to five-fold after exercise. In the liver, increased follistatin can inhibit insulin-dependent glucose production and improve glucose homeostasis. In addition, follistatin can also act as a communicator from the liver to the skeletal muscle, WAT, and pancreas. In skeletal muscle, follistatin can promote a hypertrophy phenotype [104,106]. In the pancreas, follistatin can regulate the balance of insulin and glucose secretion [106]. In WAT, follistatin can suppress insulin-dependent liposis [107,108]. ANGPTL4 is a lipid-induced factor secreted by multiple cells, including hepatocytes. Exercise stimulates the release of ANGPTL4 from the liver into the blood and reduces lipoprotein lipase activity. HSP72 is a member of the heat shock proteins and contributes to the enhancement of intracellular stress tolerance and suppression of insulin resistance. Exercise-induced HSP72 in the liver can promote fatty acid oxidation and mitochondria function. HSP72 can also secrets into skeletal muscle to enhance oxidative capacity and mitochondria function [109,110].

Exercise is reported to reduce liver cdc2-like kinase (CLK2) levels and fat deposition in high-fat diet-induced obese mice [111]. High-intensity treadmill exercise increased insulin sensitivity by activating AKT phosphorylation [112,113]. Moderate-intensity exercise decreased insulin resistance, hepatic steatosis, and weight gain in obese mice [112]. In conclusion, exercise stimulates the secretion and interaction of multiple pathways in the liver to defend against obesity.

### 3.6. Effect of Exercise on Endocrine System

Endocrine disorders usually happen in obesity. These disorders act as a risk factor for obesity or a side effect of obesity [114]. Insulin resistance and hyper insulinemia are common disorders in obesity. In peripheral tissue, insulin can stimulate glucose uptake, inhibit the release of fatty acids in adipose tissue [115], suppress the ketones in the liver, stimulate fat and glycogen deposition, and ultimately inhibit the circulating levels of all metabolic fuels. In the central nervus system, the central insulin response curbs food intake [116], promotes memory function, and improves whole-body insulin sensitivity [117,118]. Maintaining the balance of insulin is important. However, obesity leads to a progressive defect in insulin secretion and enhances insulin resistance, which could further strengthen the development of obesity. Exercise can correct the imbalance of insulin. Regular physical activity can enhance glucose uptake in muscles and promote insulin signaling in the brain [41]. Evidence demonstrates that exercise prevents inflammation, suppresses endoplasmic reticulum stress, and increases insulin signaling in the hypothalamus [119,120]. Exercise can also improve the islet beta cells’ function, stimulate the secretion of insulin, enhance insulin sensitivity, and inhibit insulin resistance.

Ghrelin is a growth hormone recretagogue [121,122,123,124] and exerts a key role in the regulation of adiposity, food intake, and metabolic diseases. Because of the close link with lipid metabolism, ghrelin has been considered as an anti-obesity target [125]. Ghrelin can control the progression of obesity by the hypothalamus. Ghrelin modulates the level of hypothalamic peptides, stimulates the neurons expressing NPY/AgRP and orexin, suppresses CRH-producing neurons, ultimately attenuates the reduction in food intake and body weight [126,127,128]. Although large studies demonstrate the relationship between exercise and ghrelin in obesity, there still are ambiguities within the studies. Additional studies need to be conducted to clarify the relationship between exercise and ghrelin and the potential mechanism [129].

Thyroid hormones regulate cellular respiration and thermogenesis, influence resting metabolic rate, and ultimately control energy expenditure [130]. Hypothyroidism decreases thermogenesis, reduces metabolic rate, and promotes the onset of obesity [131]. Exercise exerts a vital role in obesity through thyroid hormones. Swimming can balance lipid metabolism by inducing the level of thyroid hormone [132]. A single bout of exercise increases thyroid hormones levels 20 h later after exercise and further promotes lipolysis [133]. However, there are still some inconsistent results. Seven-day intense strength training can reduce thyroid levels and impair the function of the thyroid gland in elite men athletes [134]. Regimens of 24weeks or one year of strength training have no obvious effect on the level of thyroid hormone in elite athletes [135,136]. Inconsistent results between exercise and thyroid hormones may be due to exercise intervention type, or the subject type, or the duration of intervention. Additional related studies need to be conducted to figure out the relationship.

Leptin is a signal linking adipose tissue and the central nervous system. WAT-secreted leptin transmits body fat signals to the hypothalamus, activates POMC neurons [137], inhibits NPY/AgRP protein neurons, and contributes to anorexia behaviors [138]. Therefore, increasing the leptin level is considered as a target for the treatment of obesity. Exercise enhances the endocrine signals of diet and energy expenditure in the hypothalamic nerve. Swimming, voluntary running wheel exercise, and aerobic treadmill exercise were performed in the studies of leptin. Swimming or 6 weeks of voluntary roller exercise can improve hypothalamic leptin signaling and induce anorexia behaviors [139]. Watt, M. J., 2014, used a treadmill daily with the program of 18 m/min for 70 min with 5° inclination [140]. Long-term, consistent exercise training did not improve hypothalamic leptin signaling and concomitant anorexic behaviors, according to later research [95]. It is not only the time-sensitive effect of exercise on hypothalamic signals and food intake responses, but the differences of exercise protocols and hypothalamic plasticity in animals of varying ages that could also be affected.

### 3.7. Effect of Exercise on Skeletal Muscle

Skeletal muscle is the most abundant tissue in the body, accounting for 40–50% of total body weight in non-obese adults, and is a major determinant of total body energy expenditure [141]. Healthy skeletal muscle is responsible for whole-body glucose homeostasis and insulin sensitivity [142]. Dysfunctional skeletal muscle induces multiple side effects. Previous studies demonstrate that inflammation emerges in obese skeletal muscle, accompanied by increasing immune cell infiltration and pro-inflammatory activation. In addition, obesity impairs the structure and mass of skeletal muscle, promotes the accumulation of intramuscular lipids, and leads to the dysfunction of skeletal muscle [141]. Obesity also decreases the glucose uptake of skeletal muscle and leads to inflexibility of the muscle metabolism, which leads to insulin resistance.

Physical activity exerts an anti-obesity effect. Multiple studies show that exercise stimulates the secretion of myokines and maintains health. Myostatin was first discovered 25 years ago, acts as a myogenesis myokines, and regulates muscle mass [143]. Muscle hypertrophy is a phenotype in myostatin knockout mice, cattle, and dogs [144,145]. Exercise reduced the plasm and muscle myostatin level in humans [146], rats [147], and mice [148]. Apelin is another exercise-induced myokine and improves the muscle cell metabolism and contributes to a beneficial metabolic effect in obesity [149]. In both apelin global knockout and muscle-specific knockout mice, sarcopenia occurs. Apelin promotes biogenesis and mitochondria function in obesity-related sarcopenia and plays a role in the treatment of obesity. In addition to muscles, myokines can also make a cross-function to BAT, the liver, and the brain. Irisin acts as a myokine and is generated by the cutting of fibronectin type III domain-containing protein 5 [150]. In the nervous system, exercise-induced irisin can inhibit the appetite function and increase brain plasticity. In adipose tissue, irisin promotes browning and inhibits inflammation and adipogenesis. Interleukin-6 (IL-6) is the first estimated myokine which increases up to 100-fold after exercise. IL-6 exerts a metabolic regulator in paracrine, autocrine, or endocrine effects [151]. Studies shows that IL-6 stimulates the lipolysis and lipid mobilization of adipose tissue and provides the energy for muscle contraction and glucose in skeletal muscle. IL-6 is a sensor of whole-body metabolism. BDNF is a member of the neurotrophic family and recognized as a protector for the survival of neurons, the growth and remodeling of axons and dendrites, the differentiation of neurons, and synaptic plasticity [152,153]. Physical activity may boost the secretion of BDNF in muscle and further promote neurogenesis and synaptic plasticity and improve the neural network [154,155,156]. For obese subjects, 3-month AE performed on a treadmill or bicycle promoted the synthesis and storage of BNDF, stimulated BDNF to release into the target tissue associated with regulation of energy and metabolism, and induced moderate weight loss and the improvement of insulin resistance [157]. After a 12-week AE intervention, the resting serum BDNF level of obese adolescents significantly increased by 2.5 times [158]. A combination of AE and RE for six months reduced the incidence risk of diabetes in obese adolescents, and elevated serum BDNF levels may be a potential factor [159].

Exercise-induced myokines exert a vital role both in skeletal muscle and other inter-organs’ metabolism, and myokines are a potential therapeutic target for the treatment of obesity.

### 3.8. Effect of Exercise on the Immune System

Obesity is a low-grade inflammation disease. Exercise stimulates multiple anti-inflammatory signals to prevent inflammation in obesity [160]. Microglia, acting as a part of non-neuronal populations, control the progression of hypothalamic inflammation [161] Obesity activates microglia cells, promotes pro-inflammatory factors, and ultimately exacerbates the occurrence and progression of neurodegenerative diseases [162,163]. Exercise inhibits the toll-like receptor 4 (TLR4) signaling pathway and the death of neuronal apoptotic cells [18,164], ultimately reducing the inflammatory responses, hypothalamic apoptosis, and exerting a neuroprotective function [165,166,167]. Tumor necrosis factor α (TNFα) upregulates the expression of toll-like receptor 2 (TLR2) [168], activates downstream TNF receptor-associated factor 6 (TRAF6) [169], promotes the phosphorylation of C-Jun N-terminal kinase, and ultimately leads to an inflammatory response [169]. Inhibition of TLR2 is a promising therapeutic strategy for preventing obesity-mediated neuroinflammation and neurodegeneration. Treadmill exercise decreased the expression of TLR2, inhibited neuronal senescence and neuroinflammation, improved oxidative stress, and delayed cell apoptosis [18,170]. Transforming growth factor-β1 (TGF-β1) is positively correlated with body mass index (BMI), fat mass, and VO_2_ consumption [171,172,173]. The regulation of TGF-β1 signaling in peripheral and central tissues may be an innovative strategy to prevent metabolic and inflammatory diseases as well as age-related diseases [172]. Long-term swimming exercise can reduce the TGF-β1 protein and inflammatory signals of hypothalamus in middle-aged obese mice, hence controlling energy homeostasis to some degree [174]. Regular physical exercise can also suppress the circulating B cells, CD4^+^ and CD8^+^ cells, monocytes, neutrophils, and natural killer cells in obese mice, which indicates that exercise exerts anti-inflammation protection. Compared with obese sedentary mice, decreased neutrophils are induced in exercised mice, demonstrating exercise could inhibit the infiltration or recruitment of immune cells into adipose tissue. Macrophages are the dominant immune cells type in obesity and significantly lead to an inflammatory response [145]. Exercise stimulates the M1 to M2 shift in polarization and leads to an anti-inflammatory effect [175,176]. IL-6 acts as a cytokine induced by skeletal myocytes can also exerts an anti-inflammation effect in adipose tissue. Exercise stimulates IL-6 to release into circulation, acting as a stimulating factor to induce the secretion of anti-inflammatory cytokines, and exerts an anti-obesity role [145].

Although exercise attenuates inflammation, the precise exercise principle, immune cell types, and functional phenotypes regulated by exercise still need further studies to confirm. An overtraining exercise in rat taxed the immune function, leading to the excessive releasing of inflammatory cytokines and impairment of muscle. We should combine the complementary and integrative methods, using the single-cell technologies and multi-omics to figure out the precise exercise intensity, types, frequency, and effects on the immune system in obesity.

### 3.9. Effect of Exercise on the Sex Differences

Sex differences significantly influence exercise adaptations, primarily through hormonal variations, thermoregulation, and muscle fiber composition. Estrogen plays a critical role in providing women, particularly premenopausal women, with distinct metabolic advantages. Specifically, estrogen enhances mitochondrial function, promotes fat oxidation, and reduces adipose tissue inflammation, leading to improved metabolic health and insulin sensitivity relative to men [177,178,179,180,181]. These estrogen-mediated effects contribute to a higher rate of fat oxidation and greater metabolic adaptability in women, particularly during endurance-based activities.

Thermoregulatory mechanisms also differ by sex, influencing exercise performance under various environmental conditions. Women generally have a larger surface area relative to body mass, higher subcutaneous fat, and a lower absolute exercise capacity, all of which contribute to a reduced sweat response under heat stress compared to men [182,183]. Nonetheless, women maintain core body temperature effectively via more efficient evaporative cooling, which supports sustained performance in moderate-intensity endurance activities. Additionally, hormonal fluctuations during the menstrual cycle impact thermoregulation, with varying estrogen and progesterone levels altering body temperature and heat responses at different cycle phases [184]. Similar thermoregulatory changes are observed in women using oral contraceptives or postmenopausal women undergoing hormone replacement therapy [185].

Sex differences in muscle fiber composition further influence exercise capacity. Men typically have a greater proportion of Type II (fast-twitch) muscle fibers, associated with power and strength, favoring performance in short-duration, high-intensity activities such as sprinting and weightlifting. Women, in contrast, have a higher proportion of Type I (slow-twitch) fibers, which enhance endurance and support sustained, prolonged activities, such as long-distance running and cycling [186]. These muscle composition differences contribute to observed sex-specific advantages in strength-based versus endurance-based activities.

In conclusion, sex differences in hormonal profiles, thermoregulation, and muscle composition substantially affect exercise outcomes. Recognizing these distinctions emphasizes the importance of sex-specific exercise prescriptions to optimize health and performance outcomes.

## 4. Conclusions

**Figure 2:**
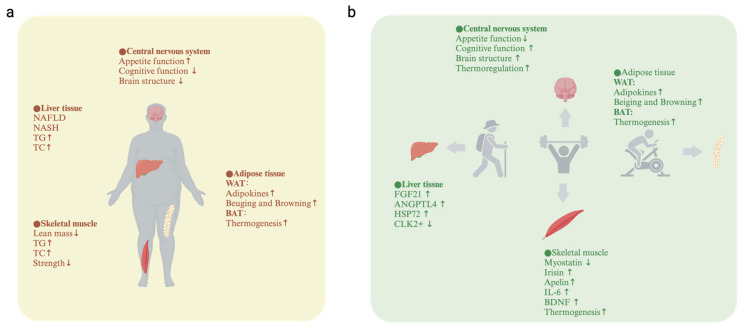
The central nervous system and peripheral metabolism in obese humans before and after exercise. (**a**) Before exercise; (**b**) after exercise. The upward arrow represents upregulation of expression; The downward arrow represents downregulation of expression. NAFLD, nonalcoholic fatty liver; NASH, nonalcoholic steatohepatitis; TG, total triglyceride; TC, total cholesterol; WAT, white adipose tissue; BAT, brown adipose tissue; FGF21, fibroblast growth factor 21; ANGPTL4, angiopoietin-like 4; HSP72, heat shock protein 72; CLK2, Cdc2-like kinase; IL-6, interleukin-6; BDNF, brain-derived neurotrophic factor “https://www.biorender.com/ (accessed on 20 September 2024)”.

**Figure 3:**
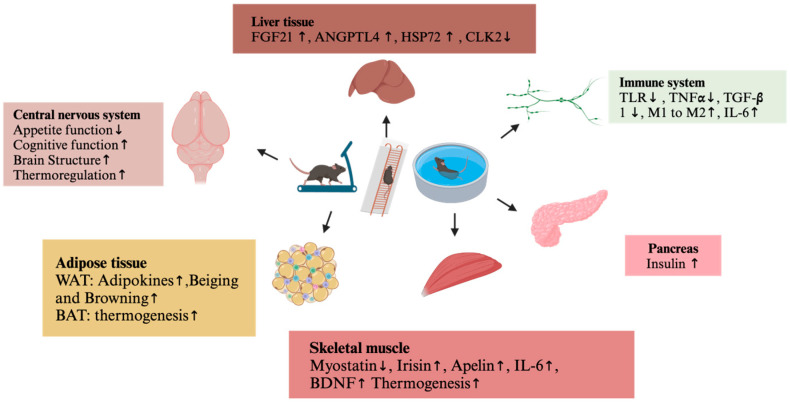
Exercise-induced changes of the central nervous system and peripheral metabolism in obese rodents. The upward arrow represents upregulation of expression; The downward arrow represents downregulation of expression. TLR, toll-like receptor; TNFα, tumor necrosis factor α; TNFβ, tumor necrosis factor β; M1, Macrophagocyte 1; M2, Macrophagocyte 2 “https://www.biorender.com/ (accessed on 20 September 2024)”.

In this review, we revealed the latest research about the effect of exercise intervention on the central nervus system and peripheral metabolism of obesity in humans, rats, and mice (Figure 2 and Figure 3). Exercise suppresses the appetite, promotes thermogenesis, increases exergy expenditure, improves brain glucose metabolism and cognitive function, and exerts a positive effect on obesity, which are consistent with the view that exercise is a medicine. In general, the underlying mechanisms might be the exercise-induced secretion of neurotrophic factors, adipocytokines, and myokines, or exercise changing the structure of the brain or adipose tissue, or exercise exerting an anti-inflammatory response and anti-oxidative stress.

Although existing studies have demonstrated numerous benefits of exercise, there are still research gaps. For example, findings on the effects of exercise on hormones, such as ghrelin and thyroid hormones, remain inconsistent, potentially due to variations in study designs and individual differences, such as age, sex, and metabolic status. Furthermore, while the short-term benefits of high-intensity exercise are well-established, further research is needed to understand the long-term effects of different types of exercise (e.g., AE, resistance training, HIIT, and moderate-intensity continuous training) on appetite regulation, weight maintenance, and metabolic health. The impact of exercise on thermoregulation, particularly the integrated control between central and peripheral systems, has not been sufficiently studied. Thermoregulation involves feedback and regulation from the central nervous system to peripheral tissues, such as the skin, fat, and muscles, which play critical roles in heat production, dissipation, and energy metabolism.

Future research should further explore how different forms of exercise optimize metabolic regulation through the coordinated action of central and peripheral systems, and gain a deeper understanding of the diverse impacts of this regulation, particularly in areas such as energy expenditure, fat oxidation, insulin sensitivity, and inflammatory response. To fully maximize the therapeutic potential of exercise in obesity management and metabolic health improvement, personalized exercise prescriptions should not only consider factors such as age, sex, and metabolic status, but also include assessments of individual metabolic phenotypes, particularly their adaptability to exercise-induced metabolic responses.

## Figures and Tables

**Table 1:** The suggested exercise interventions for obese human.

Exercise Types	Exercise Forms
Aerobic exercise (AE)	Treadmill, yoga, tai chi
Endurance exercise (EE)	High intensity of AE
High-intensity interval training (HIIT)	Combination of AE and EE

**Table 2:** The suggested exercise interventions for obese rodents.

Exercise Types	Exercise Protocols
Treadmill training	Rodents were familiarized with the treadmill for 5–7 days at 10 to 15 m/min, 30 min/day. The intensity was determined by maximal oxygen consumption (VO_2_max), maximal blood lactate, steady state, and maximal heart rate [27,33].
Resistance training	Rodents were familiarized with the climb ladders for 5–7 days before the training. In order to achieve the desired effect, ladders were often set close to the vertical of the ground (gradient 80–85%). The intensity of the exercise was determined by the number of repetitions and the load on the tail [34,35,36,37].
Swimming	Rodents were familiarized with water for 5–7 days. Water needs to be heated at about 30 degrees before the formal intervention. The protocol will be divided into low intensity (20–60 min/day, 0~3% overload) [38], moderate intensity (61–89 min/day, 0~5% overload) [39], and high intensity (≥90 min/day, ≥0% overload) [40]. Training time can also be adapted according to the aim.

## Data Availability

Not applicable.
